# Molecular Recalcitrance of Hair Passing the Digestive System of a Canid

**DOI:** 10.3390/molecules25194404

**Published:** 2020-09-25

**Authors:** Johannes Tintner, Jennifer Hatlauf, Heidi Weber, József Lanszki

**Affiliations:** 1 Institute of Physics and Materials Science, University of Natural Resources and Life Sciences, Peter Jordan Straße 82, 1190 Vienna, Austria; heidi.aut@gmail.com; 2 Institute of Wildlife Biology and Game Management, University of Natural Resources and Life Sciences, Gregor Mendel Straße 33, 1180 Vienna, Austria; jennifer.hatlauf@boku.ac.at; 3 Institute of Environmental Sciences and Nature Conservation, Kaposvár University, P.O. Box 16, 7400 Kaposvár, Hungary; lanszkij@gmail.com

**Keywords:** golden jackal (*Canis aureus* L.), animal hairs, scat analyses, FTIR spectroscopy

## Abstract

Hair is an important component in scat that is commonly used for prey analyses in carnivores. Chemically, hair predominately consists of keratin. The recalcitrant fiber protein is degraded in nature only by a few insects and soil microorganisms. Common proteases such as pepsin do not decompose keratin. Infrared spectroscopy was used to detect chemical differences caused by pretreatment and fate of hairs. Three sample sets were compared: original untreated hair, original milled hair, and hairs extracted from scats of golden jackals (*Canis aureus* L.). The results revealed that only milling affected the infrared spectral pattern, whereas digestion had no impact. Moreover, hairs from different species (e.g., boar) could be distinguished due to their spectral characteristics. They did not change through the passage of the digestive system.

## 1. Introduction

The recalcitrance of hair is an interesting phenomenon that only partly can be explained by its ecological functionality. Hairs are produced to fulfill diverse functions for our body. Due to missing repair mechanisms, certain stability and durability are required. Furthermore, hairs are replaced with a high turnover rate by shedding or molting [1]. The main chemical compound of hair is keratin, a fiber protein. Its strength is caused by the helical structure. Tensile strength clearly correlates with its diameter [2]. Microbial degradation takes place with the help of keratinolytic enzymes found in soil bacteria and fungi [3,4,5]. Only few insects are reported to consume keratin from hairs and feathers [6,7,8,9]: chewing lice (Ischnocera, Amblycera), clothes moth (Lepidoptera: Tineidae), and two beetles—carpet and keratin beetles (Coleoptera: Trogidae). Common proteases such as subtilisin, papain, and pepsin are not effective in keratin destruction. Therefore, hair is a common component in scat of carnivores.

Scat analyses are a common method in carnivore ecology research, although uncertainties and imperfect detection are frequently discussed [10]. Prey analyses of wolves (*Canis lupus*) [11,12], brown bear (*Ursus arctos*) [13], red fox (*Vulpes vulpes*), and golden jackal (*Canis aureus*) [14] but also comparative studies of different carnivores [15] are documented. The standard procedure of such analyses focuses on microscopic determination of species. Hairs are recovered from fresh scat by washing. Chemical analyses are not commonly applied.

The golden jackal is a mid-sized canid with a territory ranging from parts of Indo-China and the Indian subcontinent to Southeast Europe. Its territory spread towards northern and western parts of Europe during the last years [16,17]. Diet composition of this highly opportunistic species varies across its distribution: it comprises crabs as described from an Indian island [18], carrion of domestic animals and fruits as documented in human-disturbed habitats in Israel [19], carrion and viscera from wild ungulates in a forested area in Hungary [20], or mainly rodents in an agricultural environment as found in Bulgaria [21]. Even human remains were found in jackal stomachs related to local funeral customs in Bangladesh [22].

Fourier Transform Infrared (FTIR) spectroscopy is a powerful tool for the chemical characterization of complex matter. The infrared spectral pattern represents a fingerprint of the material. Chemical differences and changes can be assessed by this rapid and cost-efficient method. In wildlife biology, it has been introduced to study the nutrient concentration of solid rumen contents in moose [23] or diet quality of brown bear (*Ursus arctos*) [24], African elephants (*Loxodonta africana*) [25], and black grouse (*Tetrao tetrix*) [26].

The objective of this study was to determine how infrared spectral characteristics are affected by the digestive system of a canid.

## 2. Results and Discussion

### 2.1. Comparison of Original, Milled and Digested Hair

**Figure 1:**
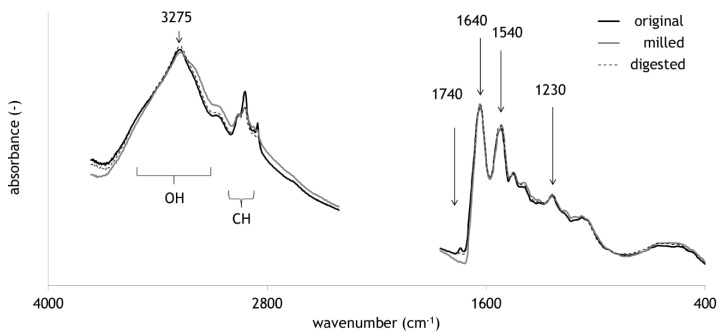
Average spectra of the three sample sets with important band regions and specific maxima.

**Figure 2:**
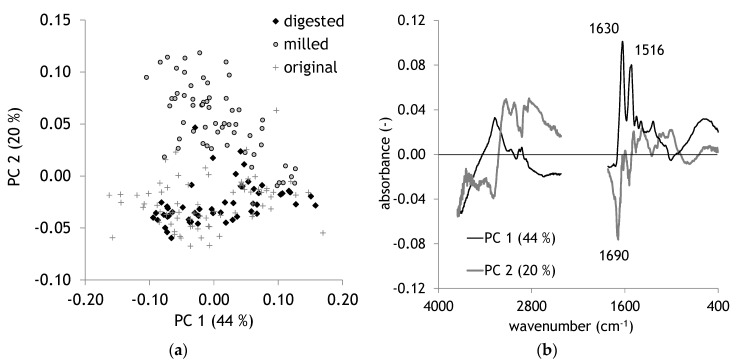
(**a**) Scores plot of Principal Component Analysis (PCA), (**b**) loadings plot, *n* = 183.

Figure 1 presents average FTIR spectra of all sample sets, original, milled, and digested. The most prominent bands are marked and described in Table 1. The band regions of OH and CH stretch vibrations are very common in most complex organic matter. Typical bands emerging in proteins are the NH stretch with a maximum around 3275 cm^−1^ and the three typical amide bands. Principal Component Analysis (PCA) was applied to compare digested hair with the other sample sets of original and milled hair. FTIR spectra revealed a separation of milled samples mainly in the second PC (Figure 2a). The loadings plot displays for PC 2 a strong negative peak at 1690 cm^−1^. PC 1 is dominated by two maxima at 1632 and 1518 cm^−1^ representing amide I and amide II bands (Figure 2b, Table 1). The effect of milling has been described for hair in Tintner et al. [27]. It was also observed for other biomolecules such as cellulose [28]. The even more interesting feature is the missing separation of digestion from original hair. Although it is well-known that common degradative enzymes like pepsin are not effective on keratin, one would expect at least certain chemical changes after a passage in a digestive system of a Canidae. Acid treatment has been documented to change FTIR spectra of hair indicated by a strong increase of the band intensity of cysteic acid at 1035 cm^−1^ [29]. A highly active bleaching treatment of wool with peroxycarboximidic acid results in the formation of cysteic acid through oxidation of cysteine [30]. In addition to chemical treatment, UV light is also able to change keratin chemistry. The formation of cysteic acid, but also alterations in the amide bands, are documented [31]. All these effects did not lead to significant changes in the presented sample sets. Milling as a hair treatment had the strongest impact on the separation of FTIR spectral information in PCA.

### 2.2. Comparison of Original and Digested Hair

**Figure 3:**
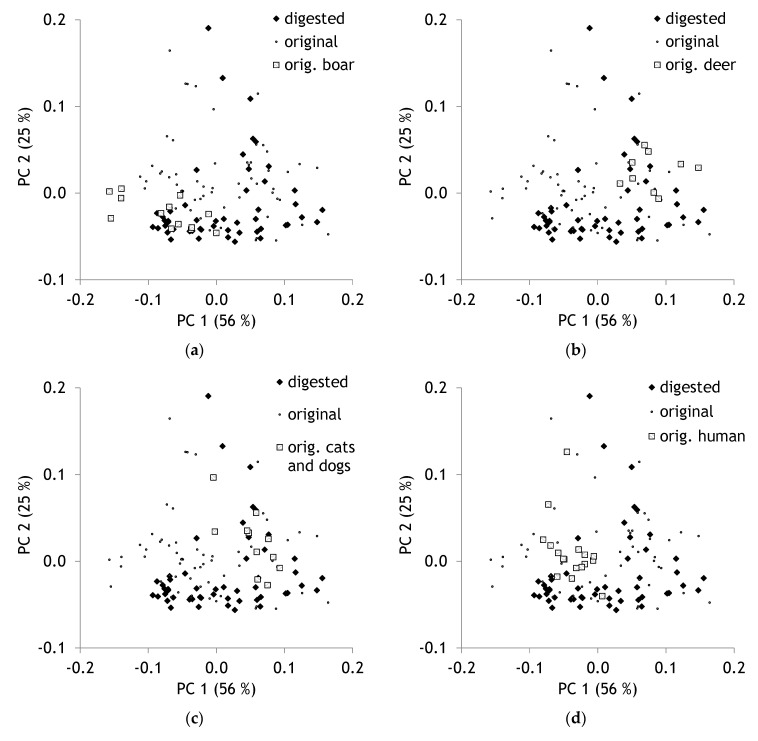

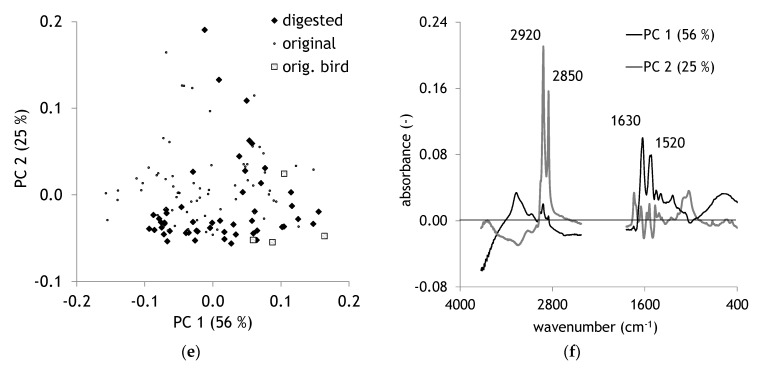
(**a**) Scores plot with highlighted samples of wild boar, (**b**) deer, (**c**) cats and dogs, (**d**) human hair, (**e**) and of bird feathers; (**f**) loadings plot of PCA containing only unground samples, *n* = 124.

For a more detailed analysis of digested hair, PCA was recalculated without milled hair samples. Differences in spectra between different species became visible. For instance, original (undigested) samples from boar hair were situated in the scores plot in the left lower corner (Figure 3a). Deer hair is situated in the same plot in the middle right part (Figure 3b), cats and dogs hair partly shifted to the left and the upper side (Figure 3c). Human hair is found in the upper left area (Figure 3d); no separation of male and female hair is visible. Bird feathers are shifted to the right lower corner (Figure 3e). PC 1 is strongly influenced by the amide I (1630 cm^−1^) and amide II (1520 cm^−1^) bands (Table 1), whereas PC 2 displays very strong and sharp maxima at 2920 and 2850 cm^−1^ (Figure 3f). These bands are assigned to aliphatic methylene groups. The results demonstrate that the chemical composition of hair differs between species. Such results are reported for elephant and giraffe hair [36]. However, it is also visible that only some species can be discriminated distinctly.

**Figure 4:**
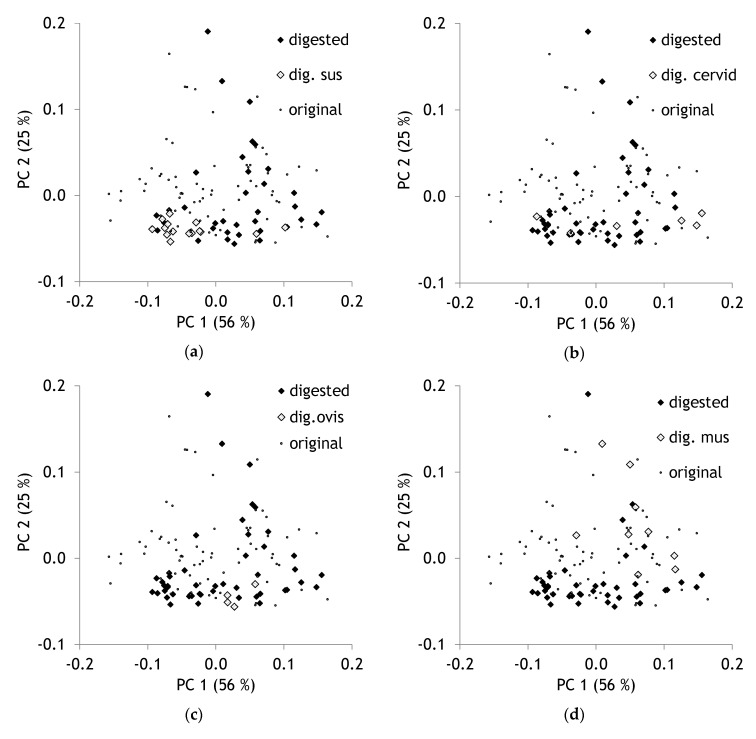
(**a**) Scores plot with highlighted hair samples after passage through the digestive tract of *Sus*, (**b**) cervids, (**c**) *Ovis*, (**d**) *Mus.*

The species pattern of digested hairs overlapped the pattern of original hair from different species. Indeed, no obvious separation of digested and undigested hairs could be observed. Species separation was found in a comparable way. Digested hair samples from *Sus* were found in the left lower corner of the scores plot intermingled with undigested hair of wild boar (Figure 4a). Cervid and *Ovis* samples were situated in the lower plot region (Figure 4b,c). Lots of digested samples were found from *Mus* (Figure 4d).

**Figure 5:**
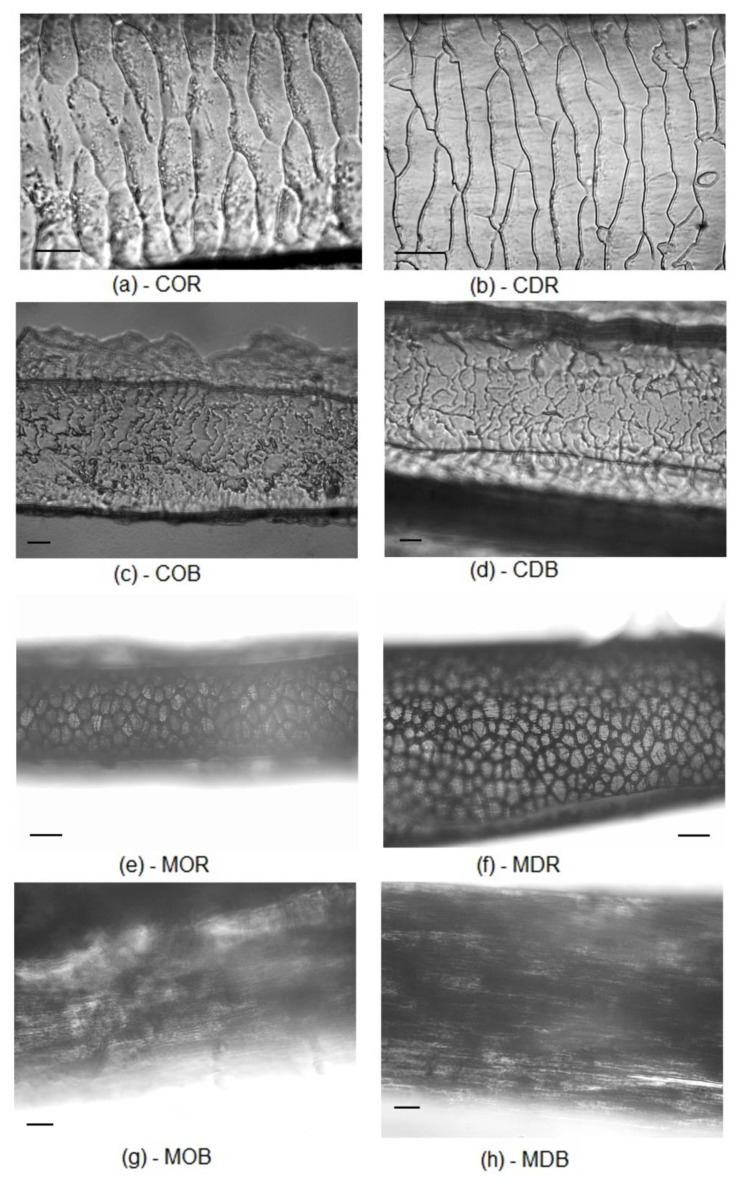
Cuticular (C) and medullar (M) pattern of original (O) and digested (D) roe deer (R; *Capreolus capreolus*) and boar (B; *Sus scrofa*) hairs; cuticular pattern: (**a**) original roe deer, (**b**) digested roe deer, (**c**) original boar, (**d**) digested boar; medullar pattern: (**e**) original roe deer, (**f**) digested roe deer, (**g**) original boar, (**h**) digested boar; scale line (**―**) on figures (**a**–**d**) and (**g**–**h**): 25 μm, on Figures (**e**–**f**): 100 μm.

Results clearly proved that the spectral patterns of hairs are more affected by species characteristics than by the passage through the digestion system of golden jackals. This fact appears astonishing as gastric acid secretion might lead to pH values of 1 [37,38]. The effect of low pH on hair structure is well-known [29,39]. As hair is used successfully in scat analyses to get information on prey composition, it is obvious that the morphological pattern is not changed considerably. Corrosion of bones in the course of digestion is reported from carnivore scats [40]. However, even plant remains contained in carnivore scats keep their anatomical pattern without remarkable damages [41]. Especially original boar (Figure 3a) and digested *Sus* hair (Figure 4a) were found intermingled with each other. Deer samples as well did not differ substantially. An apparent proof of this low level of changes due to digestion can be given by microscopic pictures of undigested and digested hair from roe deer and boar (Figure 5).

This fact would not necessarily imply that there are only negligible chemical changes. Apart from the interpretation of FTIR spectra, it must be stressed that methodological limitations must be taken into account. Only differences that can be measured by FTIR are in the focus of this study. From a theoretical point of view, the method is not capable of determining heavy metal contaminations. Such contaminations can be of certain relevance and might also change after chemical treatment. Proven measuring techniques for such questions would be atomic absorption spectrometry [42] or atomic fluorescence spectrometry [43,44].

## 3. Materials and Methods 

### 3.1. Reference Samples

To compare the hairs from scat samples with recent ones, we included a reference sample set of 130 fresh hair samples from different species. The set has been described in Tintner et al. [27]. Additionally, four reference bird feathers and two further samples were added (one human male and one horse). A compilation of the samples is given in Table 2.

### 3.2. Feeding Trials with Golden Jackal (Canis aureus L.)

The hair samples from scat in our study originated from 19 controlled feeding trials with five different golden jackal groups (comprising a total of 20 individuals). The food was chosen opportunistically based on availability in the animal parks and included mouse (*Mus musculus domesticus*), rat (*Rattus rattus*), rabbit (*Oryctolagus cuniculus* var. *domestica*), goat (*Capra aegagrus hircus*), roe deer (*Capreolus capreolus*), fallow deer (*Dama dama*), mouflon (*Ovis aries musimon*), and common quail (*Coturnix coturnix*). After a 48 h fasting period, the golden jackals were fed with the specific food. For the following 48 h, scat samples were collected twice a day and stored in a freezer. For sample preparation, the scat was soaked in water for 12 h and washed through two sieves (0.5 mm and 2 mm) to recover the remaining hair and feathers from the scat.

### 3.3. Sample Preparation

Sample preparation of the reference samples has been described in Tintner et al. [27]. A total of 59 samples were milled with a steel disc vibratory mill (Fritsch^®^ Pulverisette 9, Idar-Oberstein, Rheinland-Pfalz, Germany). Spectra of 72 samples were recorded after washing and drying at 65 °C.

A total of 52 hair samples were picked from washed scat samples. Around 20 to 40 hairs per sample were selected and dried at 65 °C.

### 3.4. Light Microscopic Hair Identification

To study the cuticular pattern of hair samples collected from jackal feeding trials, a slide was coated with a thin layer of warm 10% gelatin stock solution. The selected hair samples were placed on the surface [45]. After drying the gelatin film, the hairs were removed and the imprints were examined under a light microscope (Levenhuk 870T, Tampa, FL, USA) (magnification of 400×). To study the medullar hair pattern, hair samples were fixed on a slide with small drops of glue (colorless nail polisher). When the glue hardened, hairs were cut between the glue drops, and then hairs were mounted with paraffin oil. The medullar pattern was examined with a magnification of 100× to 400×. For hair identification, keys [45,46,47] and reference hair collection were used.

### 3.5. Fourier Transform Infrared (FTIR) Spectroscopy and Statistical Evaluation

FTIR spectra were recorded in the ATR (attenuated total reflection) mode in the mid infrared range (4000–400 cm^−1^) with an optical diamond crystal of a Bruker^®^ Helios FTIR micro sampler (Ettlingen, Baden-Württemberg, Germany) (Tensor 27). This device allows spot measurements with a spatial resolution of 250 micrometer. Thirty-two scans were recorded at a spectral resolution of 4 cm^−1^. Five replicate measurements per sample were performed. Spectra were vector normalized and the replicates averaged using the OPUS © (version 7.2) software (Ettlingen, Baden-Württemberg, Germany). Principal Component Analysis (PCA) was performed using The Unscrambler X 10.1 © Camo (Oslo, Norway). Spectral regions influenced by the diamond crystal were cut before analyses. Therefore, only band regions from 3755 to 2422 cm^−1^ and from 1817 to 400 cm^−1^ were used.

## 4. Conclusions

Our results demonstrate the high level of recalcitrance of hair during its passage through a canid digestive system. Various other factors are proven to be more relevant. Changes of the chemical fingerprint measured by infrared spectroscopy were more striking by milling than by digestion. Hairs from different species can be separated (with a certain overlap). Digestion, anyhow, neither did affect infrared spectral pattern nor did it change the microscopic appearance of hair. The results prove the high value of hair in scat analyses but are astonishing in the light of previous works proving the influence of low pH values on hair chemistry. In any case, it demonstrates impressively that proteases in the digestive systems do not decompose keratin.

## Figures and Tables

**Table 1:** Band assignment of hair spectra, wavenumbers in cm^−1^.

Band Region.	Assignment	Reference
3600–2600	OH stretch	[32]
3300–3250	NH stretch	[33]
3000–2800	CH stretch	[34]
1740–1710	C=O stretch	[28]
1640–1620	Amide I	[35]
1540–1515	Amide II	[35]
1230–1220	Amide III	[35]

**Table 2:** List of 131 reference materials.

Species	Milled	Original
human, male	14	7
human, female	20	10
goat	1	0
dog	8	6
cat	5	4
horse	5	10
donkey	2	1
cow	1	5
boar	1	13
deer	2	9
rat	0	2
badger	0	1
bird	0	4
